# The personality puzzle: a comprehensive analysis of its impact on three buying behaviors

**DOI:** 10.3389/fpsyt.2023.1179257

**Published:** 2023-08-21

**Authors:** Sibele D. Aquino, Samuel Lins

**Affiliations:** ^1^Pontifical Catholic University of Rio de Janeiro, Rio de Janeiro, Brazil; ^2^Laboratory of Research in Social Psychology, Rio de Janeiro, Brazil; ^3^Center for Psychology, Faculty of Psychology and Education Science, University of Porto, Porto, Portugal

**Keywords:** Big Five, impulsive buying, compulsive buying, panic buying, consumer psychology, COVID-19, consumer behavior, hoarding behavior

## Abstract

This study aimed at examining the role of personality traits in impulsive buying, compulsive buying, and panic buying simultaneously during the COVID-19 pandemic. At the beginning of the third confinement announced by the Portuguese government, 485 Portuguese answered in this study, mean age of 41.9 years (min = 18, max = 84; SD = 12.9), and 29.9% were men. Analyzes were carried out to investigate the association of Big Five’s personality factors with impulsive buying, compulsive buying, and panic buying. Results showed that the three buying behaviors under study have significant and positive correlations between them, and they also correlate with different personality traits. The association of each Big Five factor on buying behaviors differed. While conscientiousness was negatively and openness was positively associated with impulsive buying, conscientiousness was negatively associated with compulsive buying, agreeableness was positively associated with panic buying, and neuroticism correlated positively with all consumer behaviors. Understanding the personality traits that contribute to the development of a disorder may provide valuable insight into preventive measures and effective treatment approaches for some debilitating disorders. This study opens ways for investigating impulsive buying and compulsive buying by relating them to panic buying. It discusses the three different buying behaviors during the COVID-19 pandemic and future consumer research directions involving other variables.

## 1. Introduction

Factors such as personality traits and individual variables were relevant to investigating and predicting consumer behavior (1–3). The studies of individual differences have provided literature with diverse ways to analyze their effects. The Big Five Model of personality is, probably, the most widely used framework for explaining individual differences in populations and relies on five sharply independent traits (4). The model has also been used extensively in the study of human characteristics that have an impact on purchase behaviors [e.g., (5–12)].

Studies bespeak that personality can be defined by a set of traits that determine psychological predispositions that are stable over time. Such traits are grouped into independent dimensions, according to the Big Five personality factor model (14, 13), namely: Extroversion, which refers to how much people enjoy interpersonal contact and socializing; Conscientiousness, which is the tendency to be disciplined and regimented; Openness to new experiences, which refers to how much a person appreciates unconventional sensory and intellectual experiences; Agreeableness, which refers to how kind and cooperative a person is; and finally, the Neuroticism factor points to tendencies to demonstrate emotional instability and experience negative feelings (13).

Since these are stable individual traits throughout life and even considering cultural differences, the use of Big Five measurements is necessary for a better understanding of the influence personality traits may have on specific buying behaviors (15, 16). The Big Five model was chosen over other personality models because its widespread acceptance provides a systematic way to measure personality differences at the most basic levels (17). Prior research on consumer psychology has frequently been developed regarding the role of traits [e.g., (18,19)], the effects of hedonic and utilitarian motivation, and subjective norms [e.g., (20–22)], or the role of resources, like time and money (23).

However, individual differences also could predict consumer behavior. Not only attitudes, which are less stable ones, but mainly personality traits may be considered more stable across one’s lifespan. In this sense, identifying which personality components are involved with different buying behaviors is a useful way to build a broader understanding of regular purchases and eccentric buying, which occurs and seems to be underrepresented in the literature (15).

Above this, during a disruptive event such as a pandemic, the information overload people receive can contribute to a sense of fearfulness that increases overconsumption and thus the likelihood of product scarcity (24). Critical changes in material conditions arouse gut feelings that influence people’s behaviors (25). According to a World Bank Group report (26), the economic outcomes of the COVID-19 pandemic are severe, considering the period from the beginning turning data collection and publications about the first consumer studies during the new coronavirus spread.

During the COVID-19 pandemic, human behavior has been impacted in multiple ways, with consumption one of the most prominent aspects. As a result of the impact of the COVID-19 pandemic, consumers have exhibited a variety of behaviors. According to recent research, the global epidemic of coronavirus disease has provoked psychological distress worldwide, manifesting as pervasive feelings including distress, anxiety, and panic (27, 28). These feelings and emotions may lead to impulsive buying (29, 30), compulsive buying (31); panic buying (32); revenge buying (33).

The uncertainty raised by the health and economic challenges worldwide seems to have affected buying and other decision processes, and these facts push psychological science to understand the role psychological variables play in these behaviors. The COVID-19 pandemic is particularly a challenging environment for advances in areas where findings are not yet definitive.

Covering three different buying behaviors in the same sample during the pandemic could support important findings on personality and shopping and can extend the knowledge about shopping relative to individual tendencies and predispositions (impulsive and compulsive), as well as their relationship to a buying behavior typically impacted by the social context (panic). In this sense, this correlational study aimed to test the role of personality traits on three types of consumer behavior simultaneously (impulsive, compulsive, and panic buying) during the COVID-19 pandemic.

### 1.1. Impulsive buying

In defining impulse buying, authors tend to emphasize the spontaneity, inconsistency, and emotional state of the individual at the moment of purchase [e.g., (34–36)]. For Rook and Fisher (22), impulsive buying occurs when the consumer makes a spontaneous, unreflected, and immediate purchase. They also emphasize that impulsive buying is dominated by emotional attraction. To explain this behavior, both extrinsic and intrinsic factors could be considered. Among extrinsic factors, there are social visibility, time pressure, and economic availability. The intrinsic ones are, for example, impulsiveness and personality factors, such as neuroticism, extraversion, and conscientiousness (15, 35, 38).

Impulsiveness is considered a basic human trait (22). Therefore, buying impulsiveness should be an individual difference to be considered in studies preoccupied with understanding purchasing behavior during times of crisis (39). Impulsive buying is common among healthy individuals and reflects individual needs and differences related or not to the information to which they have access (40).

Several researchers have found associations between the Big Five personality factors and impulsive buying. Openness explains impulsive buying behavior (41), as do extraversion, achievement, and neuroticism (42). Conscientiousness negatively predicts impulsive buying, and it is also related to other individual personality factors, explained by extraversion or conscientiousness (1, 43). Specifically, neuroticism is the one that recursively comes out as the Big Five factor that is the most significant predictor of impulsive buying tendency (1, 41, 42, 44, 45). Finding new associations between neuroticism and buying behaviors may shed light on the nature of the vulnerability that high neuroticism elicits.

It is known that there are studies with results similar to each other, and others that do not corroborate previous research about the Big Five and buying behaviors. Thus, findings on personality and shopping are still not unanimous since they do not always associate with the same behaviors and are not always in the same direction. Not being consensual, it is crucial to investigate which factor influences more, or less, certain tendencies in diverse contexts.

Considering that impulsive buying is characterized by the urge to buy and stimulated by the affective state, this type of purchase behavior could sometimes surroundings on Compulsive buying–and both are consumer escapism behavior. Impulsive buying may be a signal for loss of self-control and falling into shopping addiction, so, in this aspect, it can predict compulsive buying (23, 46).

Sometimes, impulse buying looks like a synonym for compulsive buying, but it is all-important to distinguish warily these concepts. As seen, the impulse to purchase may be a simple habitual way by anyone, and a low or strong individual tendency. Distinctively, compulsive buying is chronic, and its key characteristics are a repetitive and uncontrollable desire to buy, always preceded by and resulting in negative feelings.

### 1.2. Compulsive buying (Oniomania)

Negative emotions lead to the tendency toward compulsive buying (48). When Sneath et al. (49) explored the relationship between consumer emotions and compulsive buying, they found a positive correlation between negative emotions and compulsive buying behavior. Thus, while the typical impulse buyer makes occasional spontaneous, unplanned purchases driven, most of the time, by a positive mood, compulsive buyers may employ impulsiveness and obsessive buying behaviors to cope with and alleviate their undesirable negative emotions like depression or sad feelings (50, 51). Compulsive buying is characterized by excessive shopping and buying ideas that produce distress and damage, including hard-to-control impulsivity (52). Moreover, it is essential to note that compulsive buying-shopping disorder is considered a mental disorder ICD-11 (as are other specified impulse control disorders, 6C7Y).

Evidence has demonstrated that compulsive buying may be a way of compensating for negative emotions (53), which may partially explain excessive consumption during highly uncertain social, economic, or sanitary events and the rise of general anxiety related to them. Despite sharing common traits with impulsive buying, compulsive buying may be considered rather abnormal conduct and is associated with stockpiling behaviors, characterized by the accumulation of goods and the avoidance to abandon unessential ones (54).

Recent studies have found personality factors correlated with this pathological buying behavior (11, 55, 56). Previous studies also reported moderate to high genetic correlations between neuroticism and obsessive–compulsive symptoms (Bergin et al., 2014; Taylor et al., 2011). For explaining compulsive buying, agreeableness, neuroticism, and openness are personality factors that demonstrated significant predictive power. Moreover, impulsive buying plays a mediating role in this relationship (41, 57).

The diversity of cultural contexts in which personality traits have been demonstrated to be relevant to explaining purchasing behaviors makes it reasonable to consider them as timely variables to be investigated. Understanding these variables simultaneously and not in isolation can provide a wide overview of the relationships found so far, broadening findings involving personality and three different purchase behaviors in the same context.

Compulsive buying behavior is cyclical and pathological, characterized by a repetitive and uncontrollable desire to buy, leading to negative feelings such as regret and guilt (47). This is a type of consumer behavior potentially surrounded by negative feelings, as with panic buying, for example. However, panic buying is characterized by other specific negative feelings, such as fear and panic, whose consequence is to buy beyond what is usually bought (39).

### 1.3. Panic buying

Panic buying occurs when fear and panic influence behavior, leading people to buy more items than usual (39). In previous events and during the COVID-19 pandemic, feelings of fear have been shown to elicit specific consumer behavior patterns, broadly known as panic buying, which is also related to impulsive buying (39).

Both impulsive buying and personality traits are individual differences shown to exert influence on distinct purchasing behaviors, a pattern that has been replicated with samples from different countries and during diverse major events (15, 35, 38, 41, 57). Therefore, evidence from different countries is a valuable contribution to the understanding of these phenomena.

There are other important individual differences in panic buying, like trust in government and money attitudes (15, 35). Consistent with what has been found in other cultures, levels of trust and the willingness to seek information also play a role in explaining panic buying behavior (38, 58). However, studies investigating panic buying and the Big Five are still scarce.

If personality factors may have a significant effect on consumer emotions (59), neuroticism maybe has the most significant one (60). It is generally agreed that neuroticism is the tendency to experience negative emotions such as anxiety, fear, sadness, anger, irritability, loneliness, worry, dissatisfaction, and vulnerability and that this factor is both a response to and a cause of various types of stress and diseases (61–65).

An abundance of studies indicates that neuroticism scores predict stress, psychological distress, emotional disturbance, low subjective well-being, symptoms related to physical tension, and substance abuse. Neuroticism is correlated with most depressive disorders, insomnia, schizophrenia, attention-deficit/hyperactivity disorder, anxiety, bipolar disorder, obsessive–compulsive disorder, and even cardiovascular disease (66–73). High levels of neuroticism reflect similarly high levels of stress that a person regularly experiences. So, individuals tend to behave according to a negativity bias (74) and, during the pandemic, this tendency is often associated with neuroticism scores has shown an insignificant reliance on the valence of the received information (75).

In this sense, when levels of fear, anxiety, panic, and social influence are not maintained to a given level, they may not be beneficial for consumers (76). Depression and stress were predictors of excessive shopping as a coping strategy. Excessive shopping functions as a coping strategy in times of danger, as a way for individuals to protect themselves, reduce anxiety, and alleviate negative feelings (77, 78). An exploratory analysis showed that stockpiling was associated with high scores on extraversion and neuroticism, but with low scores on conscientiousness and openness to experience (35). Behaviors such as hoarding, for example, may occur under other conditions or may be one of the symptoms of different pathologies (79). Moreover, anxiety and stress can also be a precursor to panic buying (77).

As pointed out by the literature, all three types of buying behavior have a strong emotional root. The connection between the affects and impulse buying, compulsive buying, and panic buying is signaled by most studies on each of these themes (see Table 1).

**Table 1:** Overlaps and differences between the three buying behaviors.

	Impulsive	Compulsive	Panic
Predictive emotions involved	Negative or positive (80, 81)	Negative (e.g., guilt, depression) (53, 82)	Negative (fear, uncertainty) (39, 83)
Trigger	The product (22, 84)	The behavior (54, 86)	The crisis context (39, 85)
Frequency	Occasionally (86)	Cyclical (47, 87)	Disruptive events (39)
Behavior assortment	Ordinary (86)	Pathological (54, 86)	Self-protection (88, 89)
Big Five traits associated with	Openness (41), Extraversion (42), Conscientiousness (1), Neuroticism (42)	Openness, Agreeableness, Neuroticism (11, 56)	Neuroticism (35)

Apparently, what differentiates them is whether it is commonplace behavior, casual and more impacted by positive affects (impulsive buying), whether it is a pathological and uncontrollable behavior that brings negative consequences (compulsive buying), or whether it is behavior driven mainly by challenging and disruptive events (panic buying). These aspects have relevance to the study of consumer psychology involving personality in impulsive buying, compulsive buying, and panic buying.

## 2. Study design

This correlational study aimed to test the role of personality traits on different buying behaviors (impulsive, compulsive, and panic buying) during the COVID-19 pandemic in a convenience Portuguese sample.

## 3. Method

### 3.1. Participants

In this study, there were 485 Portuguese participants, with a mean age of 41.9 years (min = 18, max = 84; SD = 12.9), 29.9% being men. The sample included people from all social classes, with 1.6% of the respondents self-reporting as lower class; 14.8% lower middle class; 65.2 middle class; 17.5% upper-middle class, and 0.8% upper class. Of the total participants, 0.6% reported primary education, 4.9% basic education, 36.1% secondary education, 41% undergraduate, 15.5% master’s, and 1.9% doctorate.

### 3.2. Instruments

An online questionnaire was used, available on the Internet. Upon agreeing to answer the survey, the participants were directed to the questionnaire that contained sociodemographic questions (gender, age, education, perceived social class). In addition to these questions, there were psychometric scales to access the Big Five, impulsive buying, compulsive buying, and panic buying to follow:

#### 3.2.1. Mini-IPIP five-factor model personality

Participants’ personality traits were measured by the Portuguese version of the Mini-IPIP [(90), adapted for European Portuguese version by Oliveira (16)]. This 20 items version aims to assess the five dimensions of personality briefly using four items for each factor. All items could be answered using a seven-point Likert-type scale, measured from 1 (totally disagree) to 7 (totally agree). Items use specific questions regarding Extraversion (*α* = 0.60; *ω* = 0.61), Agreeableness (*α* = 0.67; *ω* = 0.68), Conscientiousness (*α* = 0.56; *ω* = 0.58), Neuroticism (*α* = 0.61; ω = 0.61), and Openness (*α* = 0.62; ω = 0.64).

#### 3.2.2. Buying impulsiveness scale

It used a shortened version of the Rook and Fisher scale (22), adapted to the Portuguese context by Lins et al. (91), with four items (“Just do it” describes the way I buy things; I often buy things without thinking; “I see it, I buy it” describes me; Buy now, think about it later describes me; *α* = 0.88; ω = 0.88). The scale was measured from 1 (totally disagree) to 7 (totally agree).

#### 3.2.3. Compulsive buying scale

The Brazilian version was adjusted to European Portuguese [Faber and O’Guinn (92) version adapted for a Brazilian Portuguese version by Leite et al. (93)], two native speakers to improve the understanding. To measure shopping compulsivity, a seven-item unidimensional scale measured from 1 (totally disagree) to 7 (totally agree) was used. The items represent behaviors, motivations, and feelings associated with compulsive buying (e.g., Felt anxious or nervous on days I did not go shopping; Felt others would be horrified if they knew of my spending habits; *α* = 0.67; *ω* = 0.74).

#### 3.2.4. Panic buying scale- PBS

The original PBS is in Brazilian Portuguese (39) and was adjusted to European Portuguese by two native speakers to improve their understanding. A seven-item unifactorial scale was applied. There was the following instruction “During the current outbreak of the COVID-19 pandemic, how would you describe your buying behavior?.” For each statement, participants indicated a degree of disagreement or agreement considering recent behavior during the coronavirus pandemic, with 1 = strongly disagree and 7 = strongly agree (e.g., Fear drives me to buy more than I usually do; Panic makes me buy more things than I usually do; *α* = 0.93; *ω* = 0.94).

It is necessary to observe that the α and ω presented refer to the current data. Additionally, the score for the three types of consumer behavior was calculated using the average of the items, and higher scores indicate high levels of impulsive, compulsive, and panic buying.

### 3.3. Data collection and analysis

Participants were recruited by invitations on social networks. In terms of disclosure, this was done through social networks, specifically Facebook, Instagram, and WhatsApp. The snowball sampling method was used, in which participants are asked to share this questionnaire with other individuals who fall within the target population of the study (94, 95). The invitations explained the research and provided the link to access the questionnaire. On the first page of the questionnaire, an Informed Consent Form was available, complying with all the guidelines and standards regulating research involving human subjects in Portugal.

The questionnaires were administered between November 9th and November 30th, 2020, coinciding with the beginning of the third state of emergency decreed to contain the advance of the COVID-19 pandemic. During this period, Portugal adopted strict social measures, such as bans on driving on public roads at certain times and on certain days. In addition to banning driving on public roads between 11 p.m. and 5 a.m. on weekdays and 1 p.m. on weekends, the state of emergency included several measures to combat the pandemic, such as taking body temperature in public places such as workplaces, transportation, and commercial facilities, and requiring diagnostic testing for COVID-19 in certain situations.

In total, the research received 534 responses, but only fully completed questionnaires were used (*n* = 485). The sample size was calculated according to Dancey and Reidy (96) and Green (97) ≥104 + M, where M represents the number of predictors. The appropriate number of participants was based on the psychometric literature, which recommends a minimum of 10 respondents per item for acceptable analysis (98, 99). Thus, the sample size of this study exceeds the minimum recommended in the literature (*N* = 124 or *N* = 380).

Pearson’s r correlation analysis was performed to verify correlations between personality factors and buying behaviors. A Student’s t-test for independent samples was performed to investigate the extent to which the levels of each buying behavior differed between women and men. Bootstrapping procedures (1,000 resampling, 95% IC BCa) were performed to increase the reliability of the results, to correct for deviations from normality in the sample distribution and differences in the size of the groups, and to provide a 95% confidence interval for differences between means. Multiple linear regression analyzes (*Enter* method) tested the predictive power of the Big Five on the studied behaviors.

## 4. Results

The sample for the present study comprised 485 Portuguese who replied to all questions and all instruments. Sample characteristics are presented in Table 2.

**Table 2:** Descriptive statistics.

	*n*	%
Gender		
Men	145	29.9
Women	340	70.1
Social class		
Lower	8	1.6
Lower middle	72	14.8
Middle	316	65.2
Upper middle	85	17.5
Upper	4	0.8
Education level		
Primary	3	0.6
Basic	24	4.9
Secondary	175	36.1
Undergraduate	199	41.0
Master’s degree	75	15.5
Ph.D.	9	1.9
	**Mean**	**SD**
Age (min = 18, max = 84)	41.9	12.9
Impulsive buying	1.75	1.12
Compulsive buying	2.04	0.87
Panic buying	2.16	1.32
Extraversion	3.87	1.17
Agreeableness	5.09	0.97
Conscientiousness	5.17	0.96
Neuroticism	4.13	1.15
Openness	4.42	0.73

*N* = 485.

Initially, correlations were assessed between the five major personality factors and the three buying behaviors, as presented in Table 3. The highest negative correlation was found between conscientiousness and impulsive buying *r* (485) = −0.18, *p* < 0.01; while the highest positive correlation was found between neuroticism and panic buying *r* (485) = 0.16, *p* < 0.01.

**Table 3:** Correlations between variables.

	M	SD	1	2	3	4	5	6	7	8	9
1. Impulsive buying	1.75	1.12									
2. Compulsive buying	2.04	0.87	0.54**								
3. Panic buying	2.16	1.32	0.34**	0.33**							
4. Extraversion	3.87	1.17	0.02	−0.03	−0.11*						
5. Agreeableness	5.09	0.97	−0.02	0.05	−0.05	0.13**					
6. Conscientiousness	5.17	0.96	−0.18**	−0.16**	−0.08	0.05	0.05				
7. Neuroticism	4.13	1.15	0.09*	0.15**	0.16**	−0.10*	0.47**	−0.17**			
8. Openness	4.42	0.73	0.13**	0.09*	0.03	0.05	0.11*	−0.08	0.07		
9. Age	41.9	12.9	−0.05	−0.04	0.13**	−0.09	−0.08	0.15**	−0.14**	−0.37	
10. Perceived social class	3.01	0.65	0.07	0.00	0.10*	0.00	12*	−15**	0.00	0.10*	0.24**

*N* = 485. **p < 0.01; *p < 0.05.

A Student’s t-test for independent samples was used to determine the extent to which the levels of each purchase behavior differed between women and men. The results showed no significant differences in the scores between the genders (see Table 4).

**Table 4:** Mean differences in buying behaviors between women and men.

Variables	Women *n* = 340		Men *n* = 145		*t*-test	*p* value	Cohen’s d
	*M*	SD	*M*	SD			
Impulsive buying	1.71	1.05	1.83	1.27	*t*(483) = −1.02	0.154	−0.10
Compulsive buying	2.02	0.85	2.07	0.92	*t*(483) = −0.52	0.301	−0.05
Panic buying	2.20	1.34	2.05	1.24	*t*(483) = 1.12	0.116	0.12

A multiple linear regression analysis (*Enter* method) was conducted to investigate which five major personality factors (extroversion, agreeableness, conscientiousness, neuroticism, and openness) impacted buying behaviors. Thus, the results show that there is a significant predictive power of some personality factors on distinct buying behaviors.

While less conscientiousness and less openness predict more impulsive buying and less conscientiousness predicts more compulsive buying, less agreeableness predicts more panic buying. In a different direction, a higher neuroticism factor positively predicts all the buying behaviors in this study.

Finally, the sociodemographic characteristics were included in the regression model with the Big Five factors. Specifically, to test the effect of gender on buying behavior, as the literature reports gender differences in impulsive buying [e.g., (1, 100)], compulsive buying (101), and panic buying (39). Additional demographic variables were also examined. Some correlations between the Big Five factors and purchase behavior remain significant after controlling for demographic variables. The regression coefficients of all predictors are shown in Table 5.

**Table 5:** The predictive power of the Big Five factor on three different buying behaviors.

	Impulsive buying						Compulsive buying						Panic buying					
	*b*	*t*	Sig.	CI 95%		VIF	*b*	*t*	Sig.	CI 95%		VIF	*b*	*t*	Sig.	CI 95%		VIF
(Constant)	-	2.13	0.475	0.10	2.34			3.96	<0.001	0.89	2.65		-	2.19	0.029	0.15	2.75	
Extraversion	0.03	0.71	0.166	−0.05	0.12	1,07	−0.02	−0.39	0.696	−0.08	0.05	1.07	−0.07	1.56	0.120	−0.18	0.02	1.07
Agreeableness	−0.07	−1,39	0.001	−0.20	0.04	1,40	−0.01	−0.08	0.939	−0.10	0.09	1.40	−0.16	−3.05	0.002	−0.35	−0.08	1.40
Conscientiousness	−0.15	−3,22	0.023	−0.28	−0.07	1,09	−0.13	2.83	0.005	−0.20	−0.04	1.09	−0.07	−1.50	0.135	−0.22	0.03	1.09
Neuroticism	0.12	2,28	0.011	0.02	0.22	1,46	0.14	2.59	0.010	0.03	0.19	1.46	0.24	4.45	<0.001	0.15	0.39	1.46
Openness	0.11	2,54	0.385	0.04	0.31	1,02	0.07	1.57	0.120	−0.02	0.19	1.02	0.03	0.73	0.467	−0.10	0.21	1.02
Age	−0.04	−0.87	0.475	−0.01	0.00	1,13	−0.01	−0.30	0.764	−0.01	0.01	1.13	0.13	2.84	0.005	0.00	0.02	1.13
Gender	0.05	1,14	0.257	−0.10	0.36	1.12	0.05	1.09	0.278	−0.08	0.27	1.12	−0.07	1.48	0.142	0.06	0.07	1.12
Perceived social class	0.11	2.42	0.016	0.04	0.35	1.10	0.04	0.78	0.434	−0.07	0.17	1.10	0.12	2.60	0.010	−0.46	0.42	1.10
*R^2^*	0.07						0.05						0.09					
*ΔR^2^*	0.05						0.04						0.08					
*Cohen’s ƒ2*	0.03						0.02						0.03					
*F* (6,478)	4.25**						3.63**						6.03**					

*N* = 485. **p < 0.001.

## 5. Discussion

Personality tends to be stable in a variety of situations (14). In a state of emergency context, the role of the Big Five factors was tested to discern how they were associated with impulsive buying, compulsive buying, and panic buying. By testing the relations between personality factors and three different buying behaviors simultaneously during the COVID-19 pandemic, this study combines a perspective on personality traits and their associations with impulsive buying, compulsive buying, and panic buying in the same sample. Personality is a major determinant of consistent behavioral patterns and can interfere with various everyday situations (102, 104), and the present study indicates significant correlations between some Big Five traits and different shopping behaviors. The pandemic situation deserves highlighted just because it is a context of exceptional destabilization, not only locally but worldwide at the same time: This is a study realized during a distinct contingency.

First, regarding impulse buying, the correlations found corroborate the literature, indicating that the greater the conscientiousness–and thus the sense of responsibility and planning–the lower the individual’s tendency to buy impulsively. Similarly, the tendency to impulse buy increases as neuroticism and openness are also greater, according to previous studies (15, 45, 105). Both reactivity and emotional instability, as well as being more open-minded, imaginative, and curious, make people more prone to impulsive buying behavior.

On compulsive buying, the correlations found in this study were similar to the same personality factors correlated with impulsive buying. Oniomania tends to be higher as people are more cultured or artistically sensitive [characteristics of the openness factor (90)], and as the level of experiencing negative feelings such as anxiety and depression more strongly identified in those with higher averages of neuroticism (106, 107) is also higher. Conversely, individuals who had higher averages in conscientiousness were more likely to exhibit less compulsive buying behaviors. These results indicate that while characteristics related to intellectual and emotional sensitivity are linked to uncontrolled buying behaviors, the ability to balance planning and goal focus distances individuals from compulsive shopping.

As for panic buying, is fundamental to discuss first its correlation with impulsive buying and compulsive buying, even though both behaviors have been examined in several previous studies. Those behaviors so widely studied in other contexts have similarities, emphasized, for example, by the affects that impact each of the buying behaviors presented.

In this way, the positive correlations found between impulsive buying, compulsive buying, and panic buying showed that both individuals who tend to buy impulsively and those who are more compulsive tend to engage more in panic behaviors while shopping. We presume that anxiety about feeling in control during an unstable circumstance is also related to poor emotional regulation of unplanned purchases and to negative feelings that lead to compulsivity. These associations deserve special awareness since panic buying was a specific focal point during the COVID-19 pandemic, which provoked countless feelings of insecurity, anxiety, and fear (108–111).

More on panic buying, it also correlates positively with the neuroticism factor. It is known that individuals with higher averages in this personality factor tend to experience greater emotional instability (112, 113), which may make them more prone to feelings of insecurity and fear (116). Panic buying is preceded by fear (39), and this behavior is related to an individual’s emotional instability (114).

An apparently intriguing result is the negative correlation between panic buying with extroversion. It is presumed that this propensity to seek stimulation in interaction with others and to be active is lower when panic buying tendency increases because, to some degree, the social interactions of extroverted individuals may serve the function of appeasing the anxiety and fear driving panic buying. Even with the physical detachment imposed by the COVID-19 pandemic, it is reasonable to conjecture those individuals who seek energy in the presence of other people find ways of interacting that are sufficient to provide some armor that exempts them from engaging in panic buying.

But why would various forms of interaction with people protect them from eventual panic buying? Notably, extroversion is a characteristic, among other things, of people who enjoy social interaction (103, 104), and consequently, may be involved by blown-up strong connections and social support. Understanding that highly extroverted people more often participate in interactive and group events (115, 117), these characteristics shield such people from loneliness. A study found consumers with elevated levels of anxiety and loneliness have gotten involved in panic buying behavior during the COVID-19 pandemic (118). Thus, it is conceivable that the possible absence of loneliness in extroverts is related to the absence of anxiety and fear that would lead to panic buying. In this sense, the social interactions and high communication skills of outgoing people may provide more stability in experiencing the negative feelings that lead to panic buying. On the other hand, it cannot be assumed that people lower in the extraversion factor are compulsorily lonelier because other individual characteristics can suppress the anxiety of social isolation.

Similarly, panic buying was the only purchase behavior correlated with participants’ age. Unlike the original study that developed the panic buying scale (39), in the present study the older the individuals, the more likely they were to engage in panic buying. This is another result that is thought-provoking from a theoretical point of view and justifies being highlighted. The Big Five literature consistently indicates that emotional instability tends to decrease as people age (119–123), which was not supported by the found correlations in the present study. Thus, the fact that panic buying is more prevalent among older individuals may indicate that this consumer behavior is related both to circumstances or perceptions of current events and to the emotional instability characteristic of people high on the neuroticism factor.

The relatively high correlation between impulsive and compulsive buying deserves discussion. There may be an overlap between some characteristics of impulsive and compulsive buying, especially regarding conceptualization and observable behaviors. However, in the present study, the differential measurement was between the extent to which consumers’ buying behavior is repetitive (characteristic of compulsive buying only) or lacks impulse control (characteristic of both impulsive and compulsive buying). Some of the items on both measures may reflect the underlying tendency of consumers to be impulsive, and this may explain the higher correlation found. Lack of impulse control is an undeniable characteristic of the tendency to buy impulsively (22, 124) which may predict compulsive buying disorder (52). Thus, both behaviors are subject to similar antecedents, such as irresistible impulses to buy (23, 46).

Acknowledging some conceptual overlap, it is argued that impulsive buying may involve this uncontrollable urge to buy only once, without necessarily causing distress, while compulsive buying is a repetitive condition that interferes with personal functioning and is considered a spectrum of OCD. Other than that, it is possible to consider both impulse buying and compulsive buying are part of the same continuum and are subject to the same antecedents (101, 125). Nonetheless, each behavior is distinct from the other, and this distinction is well documented in the literature (see Table 1). Although there are certain conceptual overlaps, not everyone exhibits all three buying behaviors. Compulsive buyers, for example, may be highly impulsive and highly involved in panic buying, but not every impulsive buyer must be compulsive.

From the correlations found with the Big Five, regressions indicated the association of some personality factors on each investigated buying behavior. The models presented that personality factors predicted 5% of impulsive buying, 4% of compulsive behavior and 8% of panic buying, with some factors differing for each type of purchase. Consumer behavior is known to be multifaceted, resulting from both internal and external factors, from multiple reasons and contingencies (126–129).

So, only personality factors do not fully explain buying behavior, but they can reveal tendencies and patterns. At this point, it is essential to mention that similar studies also found low explained variance or even no impact of the Big Five factors on buying behaviors [e.g., (11, 12)]. In this sense, the current findings both corroborate previous research and add a new perspective.

Impulsive buying was predicted by openness and conscientiousness (beyond neuroticism). The positive predictive power of the openness factor can be understood by the fact that momentary impulsive buying stimuli should be more irresistible to these people who like to try new things (90). On the other hand, the negative predictive power of conscientiousness is explained by the fact that people with high averages in this factor are also more self-controlled (90) and, therefore, less likely to buy thoughtlessly. This same characteristic of people higher in conscientiousness explains the negative prediction power of this factor on compulsive buying. Consequently, if individuals with higher self-control in task performance are more disciplined and organized (90), they are likely to be “protected” from compulsive buying tendencies.

Engaging in panic buying was negatively predicted by agreeableness. This result may indicate that people with a greater tendency to show empathy, altruism, and pro-social behaviors will not tend to be gripped by the fear that drives people to buy more things than usual. Although the pandemic context is overly disruptive for everyone, the negative feelings of uncertainty do not impact people with higher mean scores of agreeableness, perhaps because these people avoid stockpiling by imagining that their excessive consumption could lead to an unnecessary shortage of products and cause a scarcity of items for their peers and their communities. Studies using an experimental approach could deeply investigate these causes in the future.

We identified that high levels of vulnerability, stress, and sensitivity predicted all of the different consumer behaviors tested. High averages of neuroticism were positive predictors of shopping compulsion, which is likely to be enhanced by frequent negative emotion stages that intervene with reasoning and decision-making ability (130). In the case of panic buying, neuroticism also was a positive predictor, strongly indicating how emotional instability and anxiety drive fear and panic that lead to excessive and dysfunctional shopping during challenging events.

The neuroticism factor is like a joker in big five’s literature, a wild card in the deck of behaviors associated with emotions. In response to various types of stress, individuals tend to experience negative emotions such as anxiety, fear, sadness, anger, guilt, disgust, irritability, loneliness, concern, self-consciousness, discontent, hostility, shame, reduced confidence, and feelings of vulnerability, and to engage in situations that promote negative affect (62, 131).

A growing body of evidence suggests that neuroticism has a profound impact on mental health (65, 132–138), as high levels of neuroticism reflect similarly high levels of distress and stress that an individual experiences on a regular basis. Thus, while neuroticism is associated with a variety of disorders, we propose that the same may be true for unusual shopping behavior. An individual’s propensity to shop may indicate that he or she has a co-occurring disorder or other difficulties with his or her mental and psychological health. Observing this may tell us as much about who we are as it does about how we shop.

Among the commonly used reliability statistics, Cronbach’s alpha has been the most frequently cited in the literature. However, consistent studies reveal that tau-equivalence is often violated (139–141). To overcome these limitations, psychometrists recommend using McDonald’s Omega (142) as the best index of internal consistency compared to other reliability indices (143–146). Accordingly, the present study presents both indices, emphasizing that McDonald’s omega is more suitable for evaluation (139, 147).

Here, it is necessary to briefly discuss some scale indices. Brief assessment measures are helpful for researchers who are faced with limited assessment time (148, 150), but this imposes some measurement challenges. The present study used instruments that are commonly used in the professional literature. Despite their convenience, such brief measures can be criticized for their psychometric quality, especially problems with low reliability (148), which is a real challenge for personality scales, for example, the BFI-10 (146, 149).

In the case of PIP, previous studies have already reported that the trait of conscientiousness has lower reliability compared to other traits (16, 151). This was also observed by Cooper et al. (152), who presented conscientiousness with indices *α* = 0.67, and by Wielkiewicz (151), whose Conscientiousness factor reached α. = 0.64. Also, in adapting the instrument to European Portuguese, the trait showed low values (*α* = 0.67) (16). These results are consistent with the results of this study, in which the trait of conscientiousness had a reliability of 0.57. However, we reiterate that although the alpha values appear unsatisfactory at first glance, it is worth noting that for social science constructs in general, only alphas below 0.50 are considered unacceptable (153). Therefore, it is understood that the results obtained are consistent but should be interpreted with caution (154).

Purchasing goods to a given satisficing threshold, which may vary from individual to individual according to the above-discussed factors, is a way to cope with uncertainty (155, 156). Thus, some kinds of buying behaviors, especially panic buying, may have compensatory roles in human functioning.

Personality is an important variable in the analysis of consumer behavior. The results of the present study, conducted in the context of the pandemic, suggest that other potential individual drivers deserve attention from psychological science. Despite the robustness of previous studies not only of consumer behavior but also of human decisions under uncertain circumstances, the factors that influence purchase and its possible consequences must be further explored, more specifically other individual traits, like impulsive buying tendency (in the present study showed a strong correlation with compulsive and panic buying). Although individual differences contribute to advancing the understanding of buying behaviors, identifying which behavioral variables and habits precede consumption trends and choices in different contexts is also a challenge for psychology.

This research does not ignore additional confounding factors that may influence purchasing behavior. For example, in the present study, we tested age, gender, and perceived social class. Age is not only correlated with panic buying but is also a predictor of it. It is assumed that people naturally take on more household and family responsibilities as they age. Therefore, the tendency to buy more than usual to guarantee high stock levels may have been a way that grown-ups to minimize other insecurities, uncertainties, and the instability of the pandemic scenario (157).

From a socioeconomic point of view, in addition to the changes in daily life, access to information was considered a prominent role (158). The quality of received information and access to data is also linked to social, educational, and economic issues (108). It is conceivable to speculate that the troubled collection data period could have increased panic buying behavior among those who stayed more connected to information that impact emotions–such as the higher social classes.

Considering that panic buying is influenced by fear and the perception of a lack of control over the future (25), this circumstance may have contributed to the growth in feelings of fear and uncertainty. Finally, although the literature has reported gender differences in impulsive buying (1, 100), compulsive buying (101), and panic buying (39), the present study did not confirm these findings.

It should be noted that learning about shopping behavior is as relevant to psychology as it is to management, public policy, and psychiatry. Critical changes in disruptive conditions tend to arouse gut feelings that influence people’s behaviors (25). This is especially true because shopping tendencies can wax and wane in intensity over time, leading to varying prevalence rates among people. Moreover, in many countries, there is insufficient awareness of buying behaviors because conclusions about human behavior are based primarily on observations from Western, Educated, Industrialized, Rich, and Democratic (WEIRD) samples, particularly from the United States (159–161). Intending to increase knowledge about various aspects of the disorder, the present study is important to increase the conceptualization and study of buying tendencies in populations other than WEIRD samples.

This study, however, was not without its limitations. First, there are limitations regarding context and sample characterization. There is not much control over this. This study’s limitations are the overrepresentation of women and people with higher education from a specific European country. At the same time, although one of the strengths of this study is the data collection during the COVID-19 pandemic, the social desirability bias for answers about making extra purchases, even stronger in times of crisis, cannot be ruled out.

Additionally, the present findings should consider other limitations. We highlight that one of them was to disregard other individual variables such as hedonic motivations, affects, or mood, whose association challenges in-depth studies on consumption, even more so when these purchase behaviors are essentially linked to emotions –such as impulsive buying, the compulsive buying, and the panic buying. Beyond this, the analysis of these phenomena would acquire strength if evaluated also with other psychological factors such as fear or social support. Another limitation was not considering online shopping habits and behaviors, which could extend our understanding of how social media use had affected consumer anxiety and consequently internet responses, where online shopping channels received great attention and greater demands during the COVID-19 pandemic [e.g., (162)].

Presumably, situational, and social variables also may impact a shift in consumer behavior, along with basic individual dispositions. The Big Five perspective is a personality trait model that has a high degree of consensus and stability, encompassing observable, environmental, and biological variables (104). Even though the literature has provided consistent evidence for the organization of personality through the Big Five (163), exclusively adopting a structural model of personality may limit studies of purchase behaviors. Other personality measurement models [e.g., (164, 165)] could enable or collaborate with further studies in Consumer Psychology. Thus, future investigations of individual differences in purchase behavior may incorporate other personality trait models–like HEXACO (164) or 3 M (165) more theoretically related to consumption, reflecting stable dispositions, but in a specific way to contexts of purchasing products or services. Future studies could also specify the clusters formed by the psychological variables based on personality models. Cluster analysis must be relevant to classify people into narrow profiles, identifying subgroups or prototypes among buyer behaviors based on their demographic characteristics, habits, and preferences.

Finally, it ought to be warned that the COVID-19 pandemic had a powerful impact worldwide. It should not be overlooked or ignored, that the fear of unknown circumstances caused substantial changes in the lives and behaviors of individuals (158), and society’s daily life changed throughout this period, triggering changes also in consumer habits (25). Cases of panic buying, excessive stockpiling, and revenge buying were reported worldwide and were not rare (166–169). The pandemic period implied a noticeable change in shopping habits around the world.

Notwithstanding the conceptual overlaps and differences, buying behaviors could be compensatory and maybe can function as a kind of coping strategy for alleviating the negative feelings caused by the COVID-19 pandemic. Fear–a powerful engine of human behavior, especially in times of crisis like the pandemic–also leads people to hoard goods and products, buying more items than they usually would (39). It is always important to note that hoarding behavior may occur under other conditions or may be a symptom of other pathologies (79).

Studies are addressing only one or two types of purchasing separately, and many of the findings are not consensual and have not been tested extensively in periods of global health and social crisis. Although corroborating previous findings, the present study is relevant also because it was carried out in a European sample. This differentiates it from most similar studies since these were mostly performed with Anglo-Saxon samples. Furthermore, the current study presents the triad behavior of consumers during a disruptive situation, which confirms the role of neuroticism as a wild card in consumer behavior.

In addition to the cited suggestions for further research, the broader implication of this study is to raise possible strategies to reduce dysfunctional buying behaviors. First, impulse buying needs that people be aware of the stimuli in the environment and their impulsivity so that this behavior does not cause any future damage. Pathological compulsive buying and panic buying need to be addressed by provoking the importance of self-awareness about people’s personality traits, preferences, and emotions.

This knowledge would give individuals some protection against compulsive and panic buying behavior. For example, even if different personality traits can respond to stress in many ways, it is known that people with high neuroticism deserve special attention from mental health professionals because of the impact of this personality trait on buying behaviors. Last and foremost, especially in times of crisis, self-awareness about emotions and feelings can provide the necessary self-control in buying and the indispensable self-reinforcement in emotional regulation. The shopping bag can contain healthy limits and balance.

## Data availability statement

The raw data supporting the conclusions of this article will be made available by the authors, without undue reservation.

## Ethics statement

Ethical review and approval was not required for the study on human participants in accordance with the local legislation and institutional requirements. The patients/participants provided their written informed consent to participate in this study.

## Author contributions

SA and SL contributed substantially to the conception and design of the study. SL organized the database. SA performed the statistical analysis and interpretation of the data and wrote the first draft and all sections of the manuscript. All authors contributed to the critical review of the manuscript and read and approved the submitted version.

## Funding

The author(s) declare that financial support was received for the research and/or publication of this article. This work was supported by national funding from the Portuguese Foundation for Science and Technology (UIDB/00050/2020).

## Conflict of interest

The authors declare that the research was conducted in the absence of any commercial or financial relationships that could be construed as a potential conflict of interest.

## Correction note

A correction has been made to this article. Details can be found at: 10.3389/fpsyt.2025.1673228.

## Publisher’s note

All claims expressed in this article are solely those of the authors and do not necessarily represent those of their affiliated organizations, or those of the publisher, the editors and the reviewers. Any product that may be evaluated in this article, or claim that may be made by its manufacturer, is not guaranteed or endorsed by the publisher.
