# Correction: The personality puzzle: a comprehensive analysis of its impact on three buying behaviors

**DOI:** 10.3389/fpsyt.2025.1673228

**Published:** 2025-08-13

**Authors:** Sibele D. Aquino, Samuel Lins

**Affiliations:** ^1^ Pontifical Catholic University of Rio de Janeiro, Rio de Janeiro, Brazil; ^2^ Laboratory of Research in Social Psychology, Rio de Janeiro, Brazil; ^3^ Center for Psychology, Faculty of Psychology and Education Science, University of Porto, Porto, Portugal

**Keywords:** Big Five, impulsive buying, compulsive buying, panic buying, consumer psychology, COVID-19, consumer behavior, hoarding behavior

The funder was erroneously omitted. The funder “Portuguese Foundation for Science and Technology (UIDB/00050/2020)” was erroneously omitted.

The original version of this article has been updated.

